# Computer-assisted 3D planned corrective osteotomies in eight malunited radius fractures

**DOI:** 10.1007/s11751-015-0234-2

**Published:** 2015-09-09

**Authors:** M. M. J. Walenkamp, R. J. O. de Muinck Keizer, J. G. G. Dobbe, G. J. Streekstra, J. C. Goslings, P. Kloen, S. D. Strackee, N. W. L. Schep

**Affiliations:** Trauma Unit, Department of Surgery, Academic Medical Centre, University of Amsterdam, room G4-137, P.O. Box 22660, 1100 DD Amsterdam, The Netherlands; Biomedical Engineering and Physics, Academic Medical Centre, University of Amsterdam, Amsterdam, The Netherlands; Department of Radiology, Academic Medical Centre, University of Amsterdam, Amsterdam, The Netherlands; Department of Orthopaedic Surgery, Academic Medical Centre, University of Amsterdam, Amsterdam, The Netherlands; Department of Plastic, Reconstructive and Hand Surgery, Academic Medical Centre, University of Amsterdam, Amsterdam, The Netherlands; Department of Surgery, Maasstad Hospital, Rotterdam, The Netherlands

**Keywords:** Malunion, Radius, Corrective osteotomy, 3D

## Abstract

In corrective osteotomy of the radius, detailed preoperative planning is essential to optimising functional outcome. However, complex malunions are not completely addressed with conventional preoperative planning. Computer-assisted preoperative planning may optimise the results of corrective osteotomy of the radius. We analysed the pre- and postoperative radiological result of computer-assisted 3D planned corrective osteotomy in a series of patients with a malunited radius and assessed postoperative function. We included eight patients aged 13–64 who underwent a computer-assisted 3D planned corrective osteotomy of the radius for the treatment of a symptomatic radius malunion. We evaluated pre- and postoperative residual malpositioning on 3D reconstructions as expressed in six positioning parameters (three displacements along and three rotations about the axes of a 3D anatomical coordinate system) and assessed postoperative wrist range of motion. In this small case series, dorsopalmar tilt was significantly improved (*p* = 0.05). Ulnoradial shift, however, increased by the correction osteotomy (6 of 8 cases, 75 %). Postoperative 3D evaluation revealed improved positioning parameters for patients in axial rotational alignment (62.5 %), radial inclination (75 %), proximodistal shift (83 %) and volodorsal shift (88 %), although the cohort was not large enough to confirm this by statistical significance. All but one patient experienced improved range of motion (88 %). Computer-assisted 3D planning ameliorates alignment of radial malunions and improves functional results in patients with a symptomatic malunion of the radius. Further development is required to improve transfer of the planned position to the intra-operative bone.

*Level of evidence* IV.

## Introduction

Malunion of a radial fracture may result in chronic pain and loss of function and occurs in around 5 % of the cases [1–3]. A corrective osteotomy for patients with a malunited radius fracture can improve wrist function and reduce stiffness and pain [4]. Previous studies showed that accuracy of the anatomical reconstruction is essential to achieving an optimal outcome [5–7]. Therefore, conscientious preoperative planning of the procedure and accurate surgical repositioning is required [1, 5]. Conventionally, planning is based on two orthogonal radiographs depicting lateral and posteroanterior views of the radius.

However, malunion of the radius commonly involves complex three-dimensional (3D) deformations in different planes, which may not be acknowledged on conventional preoperative 2D radiographs [8–12]. Two-dimensional radiographic planning does not always result in adequate restoration of alignment, as was demonstrated by a recent study performed by members of our study group [7].

A potential solution of the challenge presented by the complex deformity of radius malunions is the use of computer-assisted 3D planning techniques. With these techniques, both physical and virtual models of the deformed radius and the mirrored contralateral radius can be created. The models are used preoperatively to conceptualise the multiple planes of deformity and to preoperatively plan the osteotomy [4, 13]. Preoperative 3D planning also provides the possibility to create patient-specific cutting guides to transfer the planned osteotomy plane to the patient’s bony anatomy during surgery. Patient-specific guides for cutting or drilling have been successfully introduced before [14–16]. They have proven to enable accurate positioning of surgical instruments or implants with respect to bony anatomy. However, these studies mostly focus on functional results without properly evaluating residual postoperative malpositioning using 3D imaging techniques.

Therefore, the aim of this study was to assess whether computer-assisted 3D planning and the intra-operative use of personalised cutting guides improve the accuracy of bone alignment.

## Materials and methods

All patients who underwent a computer-assisted 3D planned corrective osteotomy of the radius for the treatment of symptomatic radius malunion between January 2009 and March 2014 were eligible for inclusion. Only patients who underwent a postoperative CT scan of both (full length) radii were included. Patients with a previous fracture of the contralateral radius were excluded.

### Preoperative planning

Preoperative planning was based on computed tomography (CT) scans of both the affected and the contralateral radius. The unaffected contralateral bone served as reference for determining malalignment. All CT scans were obtained using a Brilliance 64-channel CT scanner (Phillips Healthcare, Best, The Netherlands) reconstructed to a 3D volume with a voxel spacing of 0.45 × 0.45 × 0.45 mm. Data were imported by a dedicated application program which helps quantifying pre- and postoperative malalignment [17]. In short, the program enables segmenting the affected bone using a threshold-connected region growing algorithm that collects voxels that belong to the affected bone, followed by a binary closing algorithm to close residual gaps. A Laplacian level-set segmentation growth algorithm advances the outline towards the boundary of the bone. A polygonal mesh is finally extracted, which is used for visualisation of the bone deformity. It also serves to create a double-contour polygon by sampling the grey-level image 0.3 mm towards the inside (bright) and outside (dark) for each point of the polygonal bone model. This double-contour polygon with image grey levels assigned to each point enables efficient and accurate point-to-image registration.

**Fig. 1:**
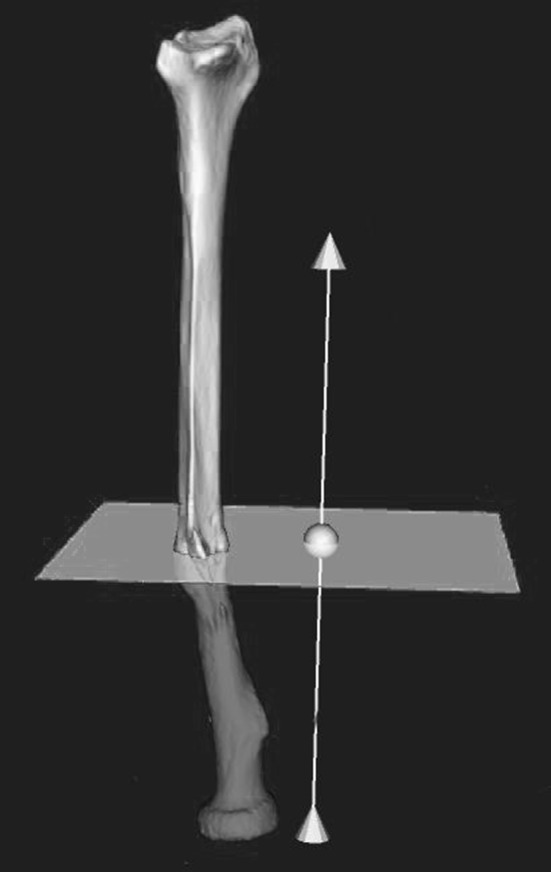
Positioning of cutting plane

**Fig. 2:**
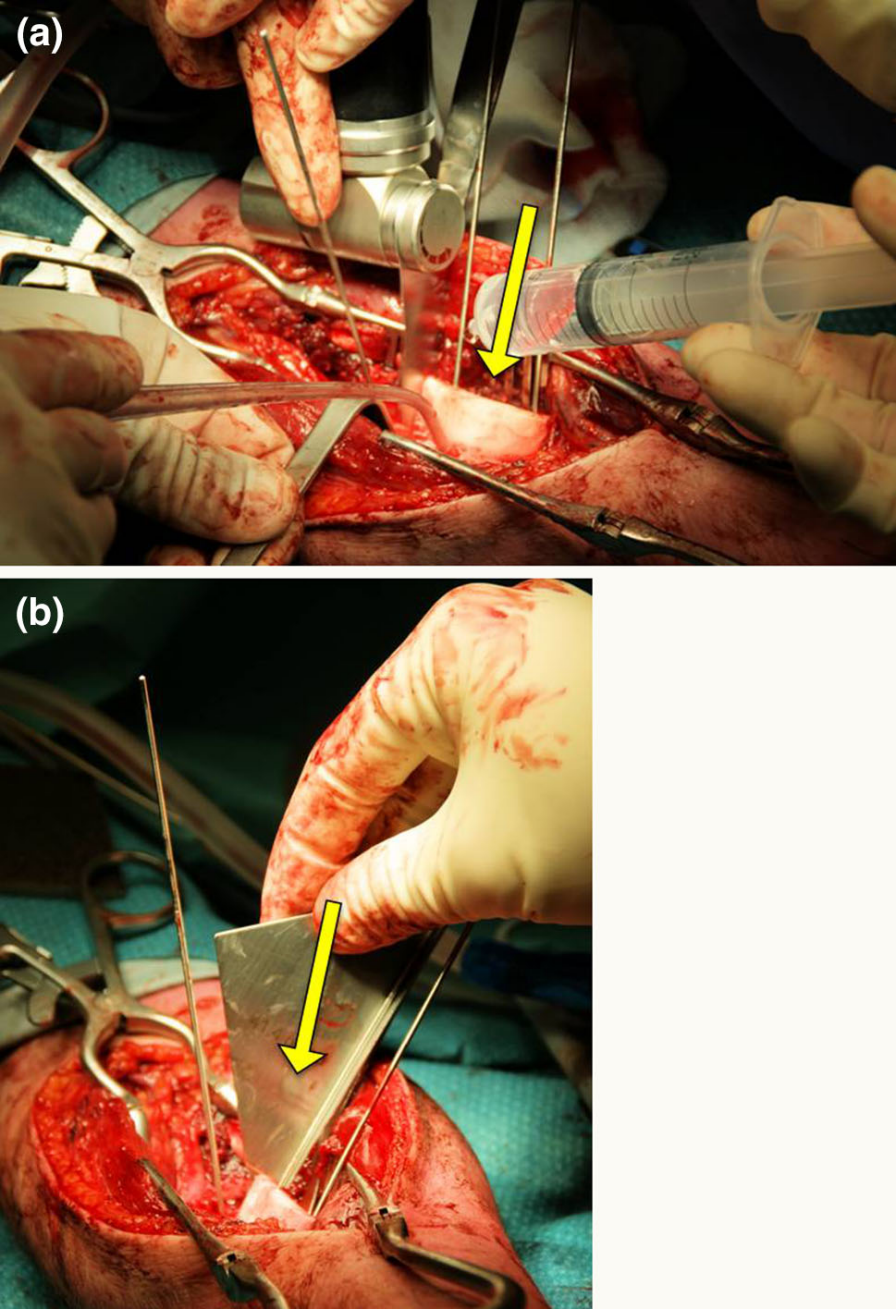
**a** Intra-operative correction of deformation with cutting guide (*yellow arrow*). **b** Intra-operative correction of deformation with angled jig (*yellow arrow*) (colour figure online)

Next, distal and proximal segments are clipped to exclude the malunited fracture region. The clipped segments are aligned with the mirrored image of the healthy contralateral bone, by point-to-image registration. This procedure provides a position matrix that brings the distal bone segment in a position that agrees with that of the mirrored contralateral bone. The matrix is used to quantify malpositioning in terms of three displacements along and three rotations about the axes of a 3D anatomical coordinate system (Fig. 4) [7]. The centroid of the clipped bone segment polygons is used as centre of rotation. Translations are determined in the ulnoradial, volodorsal and proximodistal directions. Rotations are expressed in terms of dorsopalmar tilt, radial inclination and axial rotation (pronation and supination). In case of an oblique single-cut rotation osteotomy [14], the matrix is used to determine the orientation of the osteotomy and the rotation angle for aligning the distal and proximal bone segments. The software further enables to create (1) both virtual and physical models of both radii on which the osteotomy planning was simulated (Fig. 1), and (2) patient-specific cutting guides and jigs for intra-operative guidance of the osteotomy (Fig. 2).

### Patient-specific bone models and cutting guides

During the preoperative planning, the surgeon was able to interactively set the position and orientation of the cutting plane in the virtual radius (Fig. 1). Synthetic acrylonitrile butadiene styrene (ABS) bone models were created using additive manufacturing technology (SST1200es 3D printer, Dimension Inc, Eden Prairie, MN, USA) with a resolution of 254 μm.

In four patients, a patient-specific cutting guide was used which snugly fitted to the bone geometry (see Fig. 2b). Polyamide cutting guides were manufactured (Materialise, Leuven, Belgium; Sirris, Charleroi, Belgium; Amitek Prototyping, De Meern, The Netherlands) and were sterilised before use in the operating room.

### Surgical procedure

Depending on the complexity of the malunion, patients were treated with an open-wedge osteotomy or an oblique single-cut rotation osteotomy (OSCRO) [14]. Both osteotomy types were planned by using virtual or physical synthetic models of both radii and/or assisted by intra-operative use of patient-specific cutting guides and jigs (Fig. 2). In the latter method, the sterilised surgical guide was positioned at the specific bone surface and was fixated with Kirschner wires, using the planned fixation holes. In the case of an oblique single-cut rotation osteotomy (OSCRO), the guide was removed after the osteotomy and a stainless steel jig served to set the angle between the proximal and distal bone segment [14]. Rotational alignment was achieved by rotating the malunited distal bone segment over the planned angle. Regular plate and screw fixation was performed to maintain the position. Postoperative management varied from direct mobilisation to 2 weeks of plaster of Paris immobilisation.

### Data collection and outcome

Patients were evaluated postoperatively after a minimum follow-up of 6 months. The main outcome was residual 3D malpositioning based on a postoperative CT scan of both forearms. Residual malpositioning was again expressed in terms of six positioning parameters. These residual malpositioning parameters were quantified in exactly the same way as described for preoperative planning, with the one difference that the postoperative image was used for segmentation of the bone instead of the preoperative image. Secondary outcome was the postoperative range of motion of the wrist measured on both sides with a handheld goniometer.

This study was approved by the Medical Ethical Review Committee of the Academic Medical Centre of the University of Amsterdam. All subjects gave informed consent before participation in this study.

### Statistical analysis

We reported medians and interquartile range (IQR) for nonparametric variables, and means and standard deviations (SD) for normally distributed variables. The absolute value of each malalignment parameter served to represent the residual error. The Kolmogorov–Smirnov test was used for the determination of the distribution form. The Wilcoxon signed rank test was used to compare the medians of each of the six malpositioning parameters before and after correction.

## Results

A total of 16 patients were treated for a symptomatic malunion with a computer-assisted 3D planned corrective osteotomy of the radius.

Five patients were treated recently, and their follow-up was shorter than 6 months. Two patients did not want to participate in postoperative position evaluation, and one patient had moved abroad. This resulted in a total of eight patients who were included in this series.

Of the included patients, three had originally developed a malunion after sustaining an extra-articular distal radius fracture. Five patients had sustained a forearm fracture (three antebrachial fractures and two isolated radius fractures), all of whom developed a diaphyseal malunion of the radius. The demographics of the study group are depicted in Table 1. We performed an opening-wedge osteotomy on four patients, and the other four patients received an oblique single-cut rotation osteotomy (OSCRO). All patients achieved primary osseous union. The median duration of follow-up was 26 months (IQR 12–34). No complications occurred.

**Table 1:** Demographics of study population

Case	Sex	Age^a^	Location malunion	Dominant hand affected	Indication	Technique^b^	Osteotomy type	Follow-up (months)
1	F	64	Distal, extra-articular	Yes	Pain	Cutting guide	Opening	32
2	F	53	Distal, extra-articular	Yes	Pain	Simulation	Opening	56
3	F	18	Distal, extra-articular	No	Pain, DRUJ instability	Simulation	Opening	8
4	M	32	Diaphyseal	Yes	Restricted supination	Cutting guide	OSCRO	34
5	F	18	Diaphyseal	Yes	Restricted pronation	Simulation	OSCRO	12
6	F	41	Diaphyseal + ulna	No	Restricted ROM (all directions)	Simulation	OSCRO	29
7	M	18	Diaphyseal + ulna	No	Restricted pronation/supination	Cutting guide	OSCRO	13
8	M	13	Diaphyseal + ulna	Yes	Restricted supination	Cutting guide	Opening	23

*F* female, *M* male, *ROM* range of motion, *DRUJ* distal radioulnar joint, *Opening* opening-wedge osteotomy, *OSCRO* oblique single-cut rotation osteotomy

^a^Age in years at time of surgery

^b^Technique consisted of either pre- and intra-operative simulation of the osteotomy using virtual or physical 3D models of both radii sometimes with intra-operative use of a custom-made cutting guide and angled jig

The median pre- and postoperative malalignment per dimension is depicted in Table 2. Improvement in dorsopalmar tilt showed statistical significance (*p* = 0.05, Wilcoxon signed rank test). The median residual malalignment was smallest for radial length (−0.6 mm) and axial rotation (−2.6°).

**Table 2:** Residual malalignment

Malalignment parameter	Median (IQR)			Significance^a^
	Pre-op	Post-op	Difference	
Ulnoradial shift in mm, ulnar (−), radial (+)	3.8 (1.4 to 9.9)	7.0 (1.1 to 11.0)	2.1 (−2.7 to 5.0)	0.327
Volodorsal shift in mm, volar (−), dorsal (+)	7.2 (−5.6 to 30.3)	4.0 (2.8 to 10.3)	−3.2 (−11.6 to 11.2)	0.069
Proximodistal shift in mm, shortened (−), lengthened (+)	−5.3 (−17.0 to 13.9)	−0.6 (−3.8 to 0.2)	2.9 (−0.0 to 5.4)	0.123
Dorsopalmar tilt in deg, dorsal (−), volar (−)	−9.0 (−16.8 to 13.9)	−6.4 (−7.9 to 0.4)	5.5 (−6.9 to 10.3)	**0.050**
Radial inclination in deg, ulnar (−), radial (+)	5.6 (0.4 to 8.8)	3.2 (−1.4 to 8.8)	−1.4 (−9.3 to 5.3)	0.208
Axial rotation in deg, pronation (−), supination (+)	−7.6 (−36.4 to 2.0)	−2.6 (−13.2 to 12.3)	15.0 (1.2 to −30.6)	0.484

*IQR* interquartile range, *deg* degrees, *mm* millimetre

^a^Related samples Wilcoxon signed rank test

Bold value indicates statistical significance (*p* < 0.05)

**Fig. 3:**
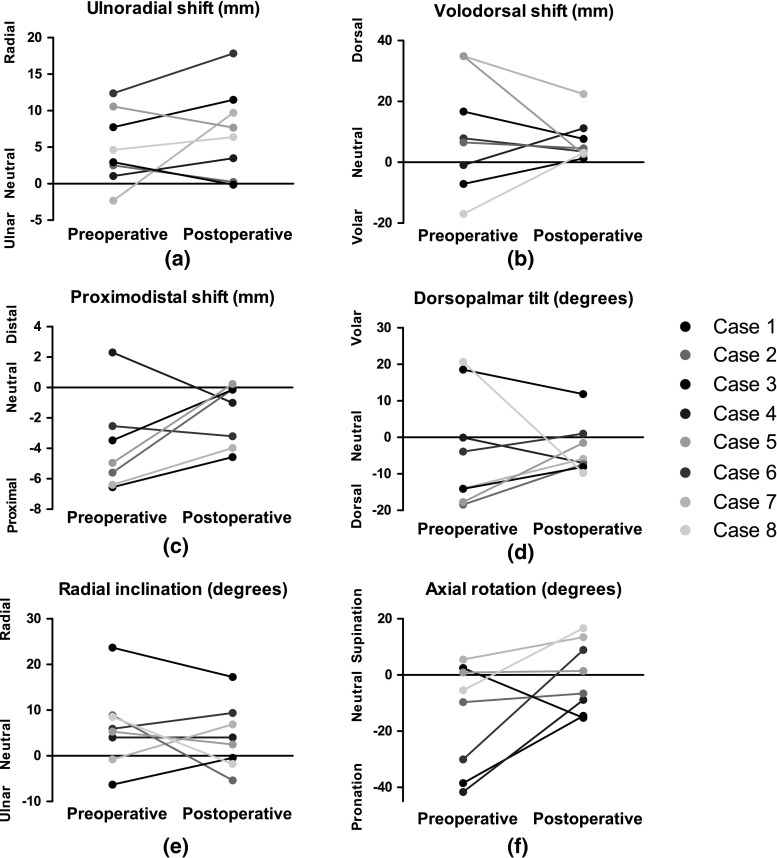
Pre- and postoperative positioning

The individual changes in preoperative and postoperative deformations are depicted in Fig. 3. In two adolescent patients (Cases 7 and 8), the radial length (translation in proximodistal direction) was not reliable due to the patients’ growing skeleton between pre- and postoperative CT scans. Volodorsal translation showed improvement (correction towards neutral) in all but one patient (88 %). In six patients (75 %), ulnoradial shift increased by the correction osteotomy. In two patients, this shift was corrected to nearly neutral.

**Fig. 4:**
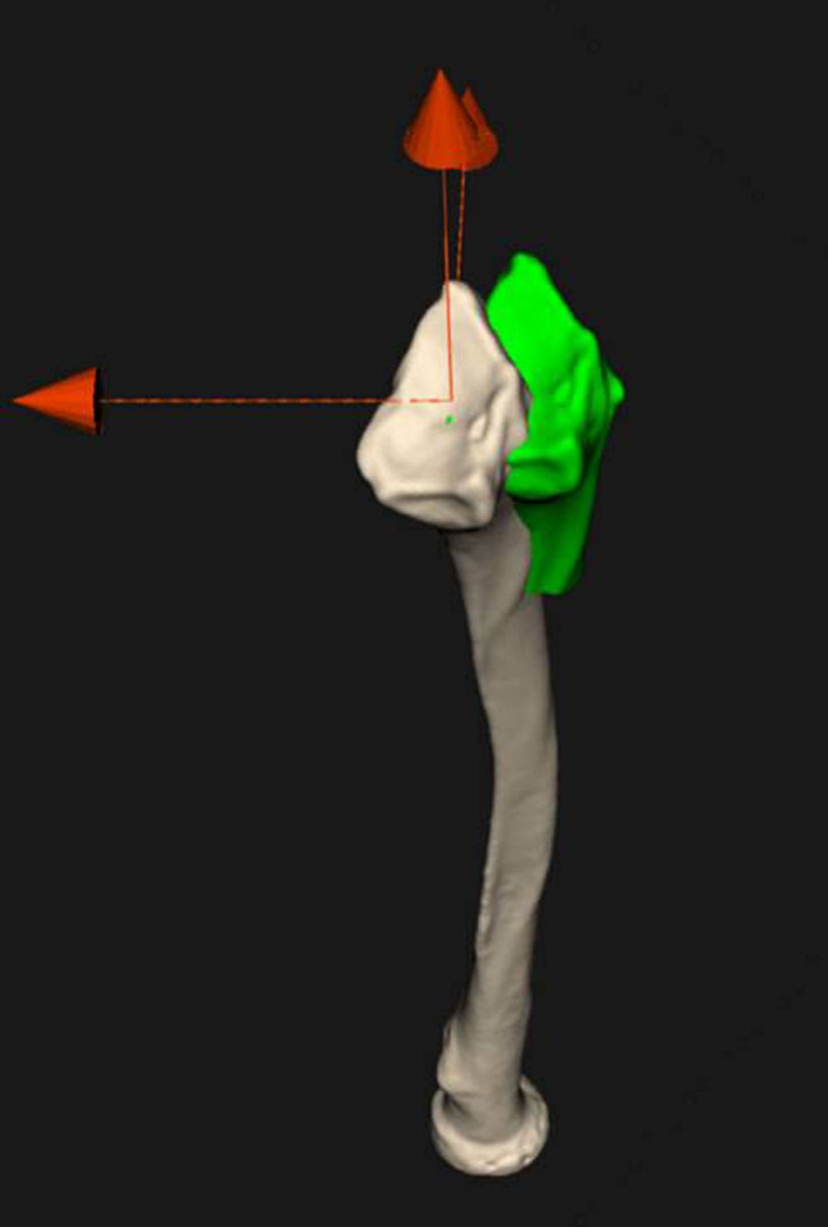
Postoperative alignment in virtual model. Postoperative malalignment of the distal radius segment (*green*) of Case 4 compared to the mirrored contralateral radius (colour figure online)

Dorsopalmar tilt was improved in seven out of eight patients (88 %): in one patient (Case 8), tilt was overcorrected from volar to dorsal. In one patient (Case 4), the preoperative neutral position was corrected to dorsal angulation (Fig. 4). Five patients originally had a malunion in pronation. In those five cases, rotations were corrected, although an overcorrection to supination was present in two patients (Cases 6 and 8). Radial inclination was improved in six out of eight patients (88 %).

Six patients (88 %) experienced a postoperative increased range of motion (Table 3). One patient (Case 3) slightly deteriorated. In addition to a distal radius fracture, this patient had sustained a triangular fibrocartilage complex (TFCC) tear that resulted in instability of the distal radioulnar joint (DRUJ). The performed correction osteotomy itself did not provide enough stability, and reinsertion of the TFCC was attempted 2 months after the corrective osteotomy, but was not successful. In one patient (Case 2), the indication for treatment was based on pain, instead of restricted ROM. The preoperative range of motion (ROM) was therefore not measured. There was no statistically significant difference in terms of malalignment parameters between the cases that were corrected with use of a cutting guide versus the corrections that were visualised (Table 4).

**Table 3:** Functional results

Case	Preoperative		Postoperative	
	Range of wrist^a^		Range of wrist^a^	
	Pronation/supination	Flexion/extension	Pronation/supination	Flexion/extension
1	150	150	165	135
2	NA	NA	180	175
3	180	155	180	150
4	115	100	145	180
5	90	NA	155	180
6	40	55	175	175
7	80	NA	135	180
8	125	180	180	180
Average	111	128	164	169

*NA* not available

^a^Expressed in degrees and measured with a handheld goniometer

**Table 4:** Differences in malalignment parameters compared to pre-op for patients treated with cutting guide versus visualisation

Malalignment parameter	Difference compared to pre-op Median (IQR)		Significance^a^
	Cutting guide (*n* = 4)	Visualisation (*n* = 4)	
Coronal shift in mm, ulnar (−), radial (+)	3.1 (1.9 to 10.0)	−2.6 (−3.0 to 3.5)	0.200
Sagittal shift in mm, volar (−), dorsal (+)	10.2 (−7.3 to 18.1)	−6.7 (−26.4 to −2.6)	0.200
Radial length in mm	2.2 (−2.0 to 15.7)	4.3 (0.3 to 5.4)	0.686
Palmar tilt in deg, dorsal (−), volar (−)	−6.8 (−24.5 to 4.4)	8.5 (5.2 to 14.9)	0.114
Radial inclination in deg, ulnar (−), radial (+)	−3.2 (−9.3 to 5.7)	0.3 (−11.4 to 5.3)	1.000
Axial rotation in deg, pronation (−), supination (+)	23.0 (11.5 to 30.6)	1.8 (−13.1 to 30.0)	0.343

*IQR* interquartile range, *deg* degrees, *mm* millimetre

^a^Independent samples Mann–Whitney *U* test

## Discussion

Postoperative 3D evaluation revealed improved positioning parameters for most patients in dorsopalmar tilt, axial rotation (pronation and supination), radial inclination, proximodistal shift and volodorsal shift. Dorsopalmar tilt significantly improved. However, ulnoradial translation was worsened by the correction osteotomy. Both over- and undercorrection occurred in individual patients. All but one patient experienced improved range of motion.

Computer-assisted 3D planning techniques are expected to optimise preoperative treatment plans and therefore minimise residual malalignment [7]. In our study, alignment improved in five of the six positioning parameters, of which improvement in dorsopalmar tilt reached significance despite the small number of patients.

There are several explanations for the residual malalignment. Firstly, the transfer from the virtual plan to the actual realignment and fixation might leave room for error. Although in half of the patients, we used patient-specific cutting jigs to transfer the planned correction onto the patients’ radius and used a jig to indicate the angle of the osteotomy, reduction and fixation were done in a freehand manner with K-wires. Although cutting guides generally show beneficial in reconstructive surgery [18], based on our results we cannot yet draw conclusions on its added value. For accurate bone repositioning in future corrective osteotomy treatment, we recommend using reduction guides [15] or patient-specific fixation plates [19].

The advantage of using an oblique single-cut rotation osteotomy is the correction of angular deformities in three dimensions while maintaining optimal bone contact. However, the method does not aim to correct translational displacements. Small rotational errors after corrective osteotomy of a diaphyseal malunion may scale to relatively large translational displacements at the distal articular level. This could partly explain the residual displacements in ulnoradial and volodorsal shifts.

Secondly, the preoperative plan does not take into account the soft tissue issues many of these deformed forearms have. Earlier (surgical) trauma often causes scar formation to structures like the interosseous membrane and makes the planned repositioning difficult to realise. Additionally, full geometric restoration of bony structures may hamper full mobility if there is too much stress on the soft tissue. Therefore, in some cases, complete correction was not obtained. Despite this issue, previously published data suggest a statistically significant correlation between residual malalignment and clinical outcome [7]. When soft tissue allows, we expect that increased precision in radiological outcome will further optimise postoperative functional results.

The strength of this study is that we examined the postoperative positioning using 3D techniques. Only a few previous studies assessed postoperative results in 3D [7, 20, 21]. However, they focussed on intra-articular distal radius malunions and expressed their findings in terms of postoperative articular displacement. Another study by Vroemen et al. [7] evaluated the postoperative malalignment in 25 patients after a 2D planned corrective osteotomy using 3D imaging techniques. The median residual malalignments we presented in this study are comparable, but not per se superior to their results after a 2D planned corrective osteotomy. However, due to the lack of preoperative 3D malpositioning of their series and a potential selection of relatively complex cases in ours, full comparison is not possible.

The postoperative range of motion we found is better than previous studies with computer-assisted 3D planned corrective osteotomy in radial malunions [22, 23]. Athwal et al. [22] included six patients with a distal radius malunion. They found an average postoperative range of motion of 89° of flexion–extension, 78 % of pronation and 74 % of supination after a mean follow-up of 25 months. Miyake et al. included 20 patients and reported a range of motion of 152° pronation and supination after a mean follow-up of 24 months.

Our functional results are also superior to published results of conventional 2D planned corrective osteotomies. A previous study that investigated the long-term results after 2D planned corrective osteotomy of distal malunions demonstrated a range of motion of 109 degrees of flexion–extension and 142° of pronation and supination after a mean follow-up of 13 years [24].

This study has several limitations. Due to the retrospective nature of this study, there was no predefined protocol for selecting patients. The decision to perform a computer-assisted 3D planned corrective osteotomy was made by the surgeon. Only patients with complex malunions were selected for this type of treatment. This approach has resulted in a selection bias and potentially limits the generalisability of our results. Due to the retrospective nature of this study, we were not able to acquire preoperative grip strength or functional questionnaires (e.g. DASH, PRWE), thus limiting the evaluation of functional outcome of the procedure. Another limitation is the heterogeneity of the population. We included subjects with both diaphyseal and extra-articular distal radius malunions. Distal malunions commonly show axial malalignment in pronation [25], whereas diaphyseal malunions typically involve angular deformation [23]. Individual cases require different goals of correction. As mentioned, an oblique single-cut rotation osteotomy (OSCRO) aims to correct rotational deformities and is limited in providing ulnoradial or volodorsal shifts. This phenomenon—in combination with the low number of cases—may explain the lack of statistically significant improvement in individual directional parameters.

Some patients may benefit more from this 3D planned osteotomy than others. Future studies should focus on determining the appropriate indication for the use of 3D planning techniques in corrective osteotomy. This study suggests that virtual 3D planning of corrective osteotomies of radial malunions ameliorates alignment. Further enhancement of this technique is required to improve transfer of the preoperatively planned position to the intra-operative bone.
